# Genome-wide association studies of *Shigella spp.* and Enteroinvasive *Escherichia coli* isolates demonstrate an absence of genetic markers for prediction of disease severity

**DOI:** 10.1186/s12864-020-6555-7

**Published:** 2020-02-10

**Authors:** Amber C. A. Hendriks, Frans A. G. Reubsaet, A. M. D. ( Mirjam) Kooistra-Smid, John W. A. Rossen, Bas E. Dutilh, Aldert L. Zomer, Maaike J. C. van den Beld, On behalf of the IBESS group, M. J. C. van den Beld, E. Warmelink, A. M. D. Kooistra-Smid, A. W. Friedrich, F. A. G. Reubsaet, D. W. Notermans, M. W. F. Petrignani, C. H. F. M. Waegemaekers, J. W. A. Rossen, A. P. van Dam, S. Svraka-Latifovic, J. J. Verweij, L. E. S. Bruijnesteijn van Coppenraet, K. Waar, M. Hermans, D. L. J. Hess, L. J. M. van Mook, M. C. Bergmans, R. R. Jansen, J. H. B. van de Bovenkamp, A. Demeulemeester, E. Reinders, C. F. M. Linssen

**Affiliations:** 1 Infectious Disease Research, Diagnostics and laboratory Surveillance, Centre for Infectious Disease Control, National Institute for Public Health and the Environment, Bilthoven, The Netherlands; 2 Department of Medical Microbiology, Certe, Groningen, the Netherlands; 3 Department of Medical Microbiology and Infection Prevention, University of Groningen, University Medical Center Groningen, Groningen, the Netherlands; 4 Theoretical Biology and Bioinformatics, Science for Life, Utrecht University, Utrecht, The Netherlands; 5 Centre for Molecular and Biomolecular Informatics, Radboud University Medical Centre, Nijmegen, The Netherlands; 6 Department of Infectious Diseases and Immunology, Faculty of Veterinary Medicine, Utrecht University, Utrecht, The Netherlands

**Keywords:** GWAS, Shigellosis, *Shigella*, EIEC, *Escherichia coli*, *E. coli*, Disease severity, Symptoms, Disease control guidelines

## Abstract

**Background:**

We investigated the association of symptoms and disease severity of shigellosis patients with genetic determinants of infecting *Shigella* and entero-invasive *Escherichia coli* (EIEC), because determinants that predict disease outcome per individual patient could be used to prioritize control measures. For this purpose, genome wide association studies (GWAS) were performed using presence or absence of single genes, combinations of genes, and k-mers. All genetic variants were derived from draft genome sequences of isolates from a multicenter cross-sectional study conducted in the Netherlands during 2016 and 2017. Clinical data of patients consisting of binary/dichotomous representation of symptoms and their calculated severity scores were also available from this study. To verify the suitability of the methods used, the genetic differences between the genera *Shigella* and *Escherichia* were used as control.

**Results:**

The isolates obtained were representative of the population structure encountered in other Western European countries. No association was found between single genes or combinations of genes and separate symptoms or disease severity scores. Our benchmark characteristic, genus, resulted in eight associated genes and > 3,000,000 k-mers, indicating adequate performance of the algorithms used.

**Conclusions:**

To conclude, using several microbial GWAS methods, genetic variants in *Shigella spp.* and EIEC that can predict specific symptoms or a more severe course of disease were not identified, suggesting that disease severity of shigellosis is dependent on other factors than the genetic variation of the infecting bacteria. Specific genes or gene fragments of isolates from patients are unsuitable to predict outcomes and cannot be used for development, prioritization and optimization of guidelines for control measures of shigellosis or infections with EIEC.

## Background

Shigellosis is caused by the gram-negative bacterium *Shigella* and can lead to dysentery [1]. The genus *Shigella* is divided in four species; *Shigella dysenteriae*, *Shigella flexneri*, *Shigella boydii*, and *Shigella sonnei*. All *Shigella spp.* are genetically closely related to *Escherichia coli* to the extent that they should be classified as one species [2, 3]. However, it is a taxonomical decision based on historical and clinical arguments that has maintained the current classification [4]. Entero-invasive *E. coli* (EIEC) is a pathotype of *E. coli*, which also can cause dysentery [5, 6]. Because of the similarity in pathogenetic features of EIEC and *Shigella spp*, differentiation using diagnostic laboratory tests is difficult [7].

As in many other countries, shigellosis is a notifiable disease in the Netherlands. This means that in each case health authorities are notified, and consequently, control measures are activated [8–11]. These control measures consist of source tracing for every shigellosis case, which places a burden on our public health system. Case definitions for shigelloses in the Dutch guidelines require confirmation with culture techniques [8]. The sensitivity of the culturing of S*higella spp.* and EIEC is low [12]. Additionally, most laboratories perform a molecular prescreening based on the *ipaH* gene, which is present in both *Shigella spp* and EIEC. From approximately half of fecal samples positive in the molecular prescreening an isolate cannot be obtained in culture [12, 13]. Shigellosis cases that are diagnosed purely by molecular procedures are not notifiable.

In contrast to cultured *Shigella spp.*, infections with EIEC are not notifiable in the Netherlands. Because of the high genetic similarities, identical disease outcomes and the low sensitivity of culturing, the two infective agents are often not detected in culture at all or are misidentified. Consequently, accurate application of the guidelines is challenging [14]. Genes of pathogens that are predictive for disease outcomes can help in the prioritization of infectious disease control measures. Moreover, the presence of genes is more easily detected by using molecular procedures as opposed to the current used culture techniques required for notification.

A few studies have investigated the association of virulence genes with disease severity for shigellosis, using Pearson’s correlation and regression analyses [15, 16]. In one of these studies, the virulence gene *sepA* was associated with abdominal pain and the combination of *sepA*, *sigA* and *ial* genes with bloody stools [16]. Another study found that detection of the *sen* (shET-2) gene was associated with diarrhea and the *virA* gene was associated with fever [15]. Both studies had a limited sample number, did not correct for multiple testing, and in one study the presence of virulence genes was established using direct detection in fecal samples. This approach is problematic, because different *Enterobacteriaceae* present in fecal samples may carry these genes, for example, on average, 2–3 *E. coli* strains are detected in the feces of a single person [17]. Therefore, assessment of single isolates would be more appropriate. Furthermore, the association with only a limited number of targeted virulence genes was conducted in these previous studies, while genomic approaches would analyze all harbored genes, gene variants, or other genetic content.

The purpose of our study is to investigate whether there is an association between symptoms and disease severity of the patients and genetic determinants of infecting *Shigella* and EIEC isolates in the Netherlands. To address this, microbial genome-wide association methods (GWAS) were applied. We hypothesize that genetic variants associated with symptoms or severity of disease allow development of specific molecular diagnostics that could predict the disease outcome per individual patient and prioritize the employment of control measures for infections with *Shigella spp* and EIEC.

## Results

### Data preparation and exploration

To assess whether other pathogens present in the fecal samples caused the symptoms and severity of patients, presence of symptoms and severity scores of patients with coinfection were compared to those of patients without coinfection. In 15.5% of the patients, a coinfection was detected. The symptom blood in stool, known as a typical symptom of shigellosis [18], was significantly less present in patients with a coinfection (chi-square, *p* = 0.019), while the presence of other symptoms was not statistically different (chi-square, *p* > 0.05). The lower fraction of patients with coinfection that experienced blood in stool was also reflected in the de Wit severity score, in which blood in stool is a criterion with double weighing, as it was significantly lower for patients with coinfection (T-test, *p* = 0.017). The Modified Vesikari Score (MVS), in which blood in stool is not a considered factor, showed no significant difference between patients with and patients without coinfection (T-test, *p* = 0.076).

The assemblies of 277 isolates were used to construct a gene presence/absence table and k-mers of variable length. This resulted in a gene presence/absence table consisting of 2890 core genes (i.e. present in all 277 isolates) and 9869 genes in total. K-mer counting yielded 28,551,795 genetic variants.

**Fig. 1:**
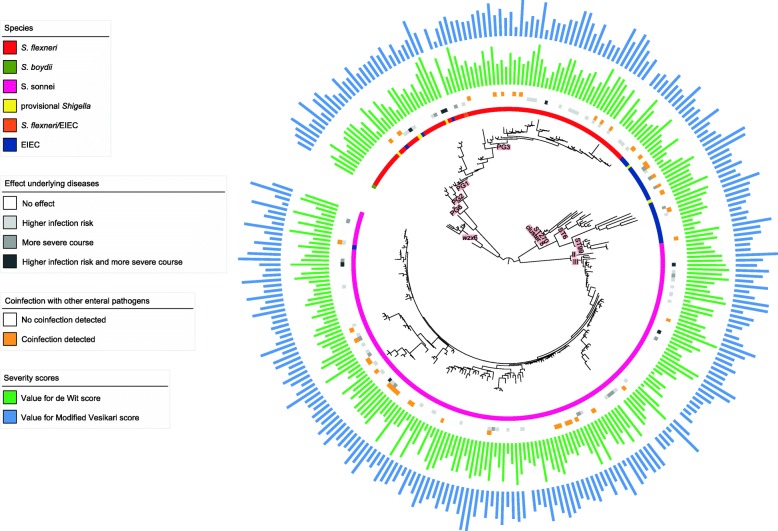
Phylogenetic tree based on core genome SNPs with species indication, underlying diseases and severity scores. Within the salmon squares are the main lineages or phylogroups depicted. wzx6 = S. flexneri serotype 6. PGx = phylogenetic group of S. flexneri. STxxx = Warwick sequence type of EIEC. II and III = S. sonnei lineage II and III

A phylogenetic tree was created based on the core genome SNPs, and the distribution of the severity scores, coinfection and the effects of underlying diseases were visualized (Fig. 1). The core SNP analysis resulted in some species-specific clusters. However, clusters that contain multiple species were also present (Fig. 1). In addition, severity scores, effects of underlying diseases and coinfection were randomly distributed over the isolates in the tree (Fig. 1). For the GWAS analysis, only isolates sequenced during this study and displayed in Fig. 1 were used. However, for contextualization of the position of the isolates in this study compared to the global population structure of *Shigella spp.* and EIEC, an additional tree was inferred including genomes from each of the main lineages and phylogenetic groups (Additional file 1). It showed that the population structure of our EIEC isolates was mainly concentrated in three clusters containing ST270, ST6 and ST99 based on isolates from the United Kingdom (UK) [19]. The UK ST270 cluster corresponded with cluster 8, the large EIEC cluster from Pettengill et al. [3]. In our analysis, EIEC isolates belonging to cluster 4, EIEC small or cluster 7, the EIEC/EHEC/EAEC cluster were not included [3]. For *S. flexneri*, a few isolates related to travel to Asia belonged to PG6 and PG2 (Fig. 1 and Additional file 1). However, the majority of isolates were PG3, consisting solely of isolates with serotype 2a or Y, and PG1, consisting of isolates of serotypes 1a, 1b, 1c, Yv and 4av. For *S. sonnei*, almost all isolates were of lineage III, only a few isolates within lineage II were detected (Fig. 1 and Additional file 1). The presence of large clusters of EIEC isolates, the presence and distribution of serotypes over the PGs for *S. flexneri* and the predominance of *S. sonnei* lineage III were described before, and are representative of population structures found in other western European countries [19–22].

### GWAS using gene presence/absence of single genes

None of the tested symptoms and severity scales resulted in significantly associated genes with a sensitivity and specificity above 85%. However, eight significantly associated genes were found with sensitivity above 92% and a specificity of 87% for the characteristic “genus”, that was used as a benchmark to evaluate algorithm performance. The gene with the highest association, produces a hypothetical protein and had a Benjamini Hochberg corrected *p*-value of 7.01E-27 and a sensitivity and specificity of 99 and 87%, respectively.

**Fig. 2:**
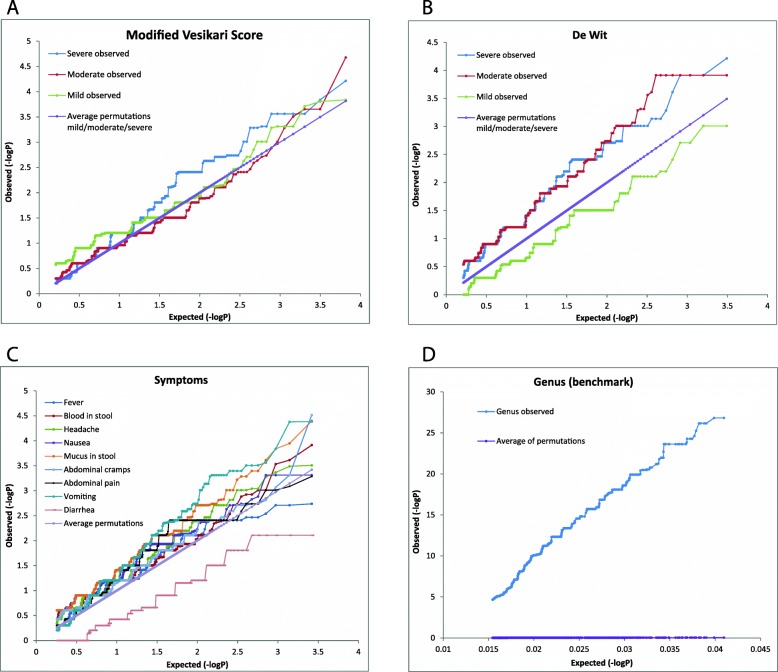
Results of Scoary: the expected versus the observed log transformed *p*-values**.** Lilac lines indicate the outcomes of the permutation dataset. **a**. Best comparison test for association of gene presence/absence with de Wit severity score. **b**. Best comparison test for association of gene presence/absence with Modified Vesikari score. **c**. Best comparison test for association of gene presence/absence with symptoms. **d**. Benjamini Hochberg’s test for association of gene presence/absence with genus

Additionally, the *p*-values of all characteristics were compared to random permutation datasets by plotting the log transformed expected and observed *p*-values against each other (Fig. 2). The gene associations with the tested severity scales (Fig. 2a and b) and symptoms (Fig. 2c) displayed similar plots as the random permutation datasets, indicating a performance as random cases. This did not apply to the benchmark characteristic “genus”, that plot showed a clear difference between expected and observed *p*-values, which was supported by the low Benjamini Hochberg corrected p-values (Fig. 2d).

It followed from the sensitivity analysis based on the benchmark characteristic “genus” that genes present in 0.7% of total isolates within the smallest group (*Escherichia*, *n* = 30), corresponding to two isolates of the total number of isolates, resulted in significant p-values. This indicated that a gene presence in a minimum of two isolates from the smallest group was enough to detect significance, if these genes were not present in the other larger group (Additional file 2).

### GWAS using gene presence/absence of multiple genes

The generated random forest model, created using isolates from the training set resulted in an out-of-bag (OOB) estimate of error rates when testing the isolates from the test set. A random error rate of 66.7% for the severity scores and 50% for the symptoms and genus was expected, as respectively three and two classes were predicted. OOB error rates in the created random forest models using 5000 trees for the prediction of symptoms and severity scales of patients were as expected for random datasets when applied to the test set. Error rates ranged from 40.8 to 53.1% for all symptoms and 65.1 to 70.1% for the two severity scales (Table 1). The construction of additional trees did not lead to better predicting models.

**Table 1:** Results of Random Forest classification and k-mer association

Characteristic	Random Forest	K-mer association with Pyseer	
	OOB error rate	No. of k-mers	Lowest LRT p-value
MVS severity scale	70.1%	0	NA
De Wit severity scale	65.1%	17	0.015
Abdominal cramps	52.7%	0	NA
Abdominal pain	40.8%	0	NA
Blood in stool	41.2%	0	NA
Diarrhea	51.6%	156	0.313
Fever	47.7%	0	NA
Headache	46.6%	0	NA
Mucus in stool	43.3%	0	NA
Nausea	53.1%	0	NA
Vomiting	51.6%	0	NA
Genus	15.9%	3,036,507	1.94E-153

In contrast, the OOB error rate of the model that predicted the benchmark characteristic genus was 15.9%, much lower than the random expected error rate of 50% (Table 1). The created model for genus prediction was further explored by examining the location of the misclassified isolates in the phylogenetic tree (Fig. 1). Comparing them with the traditional laboratory results that were obtained during the IBESS-study showed that six out of ten discrepant isolates were so-called hybrid isolates and also had an uncertain assignment using the traditional laboratory tests (Table 2).

**Table 2:** Comparison of misclassified isolates with Random Forest to traditional laboratory testing

Isolate	Phenotype^a^	Random Forest (RF)^a^	Votes^b^	Location in SNP tree	Serotype *Shigella/E. coli (agglutination)*	Properties against RF classification
IBESS811	E	S	0.99	Within *S. sonnei*	*S. sonnei* phase 1/ O-negative	Motility
IBESS97	E	S	0.80	Within *S. flexneri*	*S. flexneri*, inconclusive/ O135	Inconclusive Shigella serotype
IBESS1163	E	S	0.76	Within *S. flexneri*	*S. flexneri*, inconclusive/ O135	Inconclusive Shigella serotype
IBESS911	E	S	0.68	Within *S. flexneri*	*S. flexneri*, inconclusive/ O135	Inconclusive Shigella serotype
IBESS996	S	E	0.53	Within EIEC / *S. flexneri*	*S. flexneri* 3a/ O135	None, hybrid isolate^d^
IBESS988	S	E	0.56	Within EIEC / *S. flexneri*	*S. flexneri* 3b/ O135	None, hybrid isolate^d^
IBESS419	S	E	0.57	Within *S. flexneri*	Provisional/O-negative	None, hybrid isolate, provisional *Shigella*^d^
IBESS232	S	E	0.60	Within *S. flexneri*	Provisional/O-negative	None, hybrid isolate, provisional *Shigella*^d^
IBESS470	S	E	0.82	Within EIEC	Provisional/O-negative	None, hybrid isolate, provisional *Shigella*^d^
IBESS810	S	E	0.89	Within EIEC	Auto agglutinable^c^	None, hybrid isolate, provisional *Shigella*^d^

*RF* Random Forest*.* ^*a*^*E* Escherchia*, S* Shigella*.* ^b^fraction of votes for classification in Random Forest*.* ^c^In-silico serotype, using *E. coli serotype*Finder 2.0 of the Center for Genomic Epidemiology [23]*:* provisional/O-negative. ^d^ *Hybrid isolates* Isolates that possess characteristics of both *Shigella spp. and E. coli.*

### GWAS using k-mers

Associating k-mers with different characteristics using Pyseer did not lead to any significant k-mers for abdominal pain, abdominal cramps, blood in stool, fever, headache, mucus in stool, nausea, vomiting, and the severity score of MVS (Table 1). In contrast, 156 k-mers were associated with diarrhea, however, all k-mers had an invalid chi squared test and likelihood-ratio test (LRT) *p*-values higher than 0.313. The de Wit severity score resulted in 17 associated k- mers, whereof 15 k-mers with an LRT *p*-value lower than 0.05. An assembly of these 15 k-mers resulted in a single consensus sequence of 100 bp, based on overlapping k-mers. A BLASTn search of the consensus sequence against the database of the National Center for Biotechnology Information (NCBI, Bethesda, USA) revealed that the significant k-mers are located between two genes (Additional file 3), including a type II toxin-antitoxin gene (AYE47152.1) and a gene coding for DUF1391 (AYE48123.1), a protein of unknown function. A potential promoter region in the k-mer was found with a − 10 box (CATTATTTT) at position 58 and a − 35 box (TTGACG) at position 36 of the sequence (Additional file 3).

**Fig. 3:**
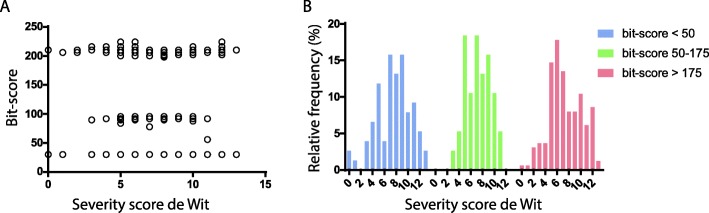
Blast result of k-mers resulting consensus on used isolates. **a**. Blast results versus severity score. **b**. Histogram of the relative frequency of the severity scores in the dataset versus the severity score of de Wit, displayed for three bit-score categories

To validate the potential of the k-mer to predict the severity score of de Wit scale, the k-mer was queried by BLAST against a database with all isolate assemblies from our study. For every sample, the bit-score of the best scoring hit was plotted against the corresponding severity score (Fig. 3a). Roughly, three groups resulted, one with a bit-score of > 175 corresponding with a full-length match with the k-mer, one with a bit-score of 50–175 corresponding to a partial match and < 50 corresponding to no match. Subsequently, the Kruskal-Wallis test was performed to investigate the difference in the de Wit severity score between the groups (Fig. 3b). No statistically significant difference between the groups was found, with a *p*-value of 0.6.

To check the suitability of the Pyseer method for the association of k-mers with characteristics in our data-set, the benchmark characteristic “genus” was used and resulted in 3,036,507 potential associated k-mers.

## Discussion

The purpose of our study was to investigate associations between genetic determinants of infecting *Shigella spp.* and EIEC isolates and the symptoms and disease severity of the patients. If such associating genetic determinants were found, diagnostics could be developed that predict the severity of the resulting disease. Additionally, it could guide prioritization and optimization of infectious disease control measures regarding shigellosis. In the Netherlands, the severity predicting capabilities of genes of other pathogens have been used previously in prioritization of control measures. In 2016, case definitions for Shiga producing *E. coli* (STEC), another pathotype of *E. coli*, were extended from culture confirmation alone to the detection of STEC by Polymerase Chain Reaction (PCR) targeting the *stx*_1_ and *stx*_2_ genes and particular virulence genes. These combination of genes within STEC bacteria are known to have associations with a higher risk for severe disease and clinical complications [24].

However, for *Shigella spp.* and EIEC in the present study, the association of the presence or absence of single genes resulted in no statistically significant association between genes with specific symptoms or severity scores with high sensitivity and specificity. Second, the association of multiple genes resulted again in no statistically significant association with specific symptoms and severity scores of patients, indicating that no complex genetic interactions that may explain disease severity could be found. Third, the association of k-mers resulted in a consensus sequence consisting of multiple aligned k-mers that was associated with a high severity score of de Wit. The sequence of 100 bp, containing multiple associated k-mers, was located between two genes with a putative promoter region with an optimal inter-base distance of 16 bases but an unclear TATAAT box. When blasting the consensus k-mer against all assemblies, three difference bit scores were observed, suggesting there are three different genetic variants of this locus. Performing a Kruskal-Wallis test on these three different bit score groups, showed that the k-mer was not valid (*p* = 0.6), and presumably was a false positive.

In our study, the genes that were associated with specific symptoms in earlier studies [15, 16], were not confirmed. In another study that was conducted in Brazil among children with shigellosis, *sepA* was associated with abdominal pain, and the combination of *sepA*, *sigA* and *ial* genes with bloody diarrhea [16]. However, it is not clear if univariate or multivariate testing for virulence genes was performed. In another study from Brazil, a case-control study was conducted. They found that the *sen* (shET-2) gene was associated with diarrhea in children in general, but not with specific symptoms of shigellosis patients. They associated the *virA* gene with fever in children with shigellosis, however *virA* was also found in 44% of controls [15]. In our study, we have used a larger sample size consisting of patients with other demographics in another setting, analyzed all genes harbored instead of a predefined selection, used other methods with higher resolution as it was based on whole genomes, and included correction for multiple testing.

Because all algorithms used in our study generated negative results for association, the characteristic “genus” was also tested as a benchmark. The algorithms used performed adequate, as they resulted in relevant genetic variants. Furthermore, a sensitivity analysis indicated that the group distribution of the characteristic “genus” was suitable for significant detection of associated single genes. This characteristic had an adverse unequal group distribution of 10% versus 90%, indicating that the number of isolates and the distribution over the groups was suitable for associating genetic content with all symptoms and severity, except for “diarrhea”, which was the only characteristic with a more unequal group distribution than “genus”. Moreover, other studies found genetic variants significantly associated with their tested traits using the microbial GWAS methods that were used in our study [25–29].

Using Scoary, single genes that had association with the characteristic “genus” were found, with low *p*-values and high sensitivity and specificity. Further, with Pyseer, over 3,000,000 potentially associated k-mers were found. This is in concordance with another study that demonstrated the suitability of k-mers for identification of *Shigella spp.* and *E. coli* isolates based on whole genome sequences [30]. Moreover, using Random Forest, OOB estimate error rate for the benchmark characteristic “genus” was 15.9%. This indicated that the model that predicts the genus of unknown isolates performed better than random, however, it does not accurately predict the genus of some isolates. Notably, six out of ten discrepant isolates also had an uncertain assignment with traditional laboratory tests. If we exclude these isolates, the OOB estimate error rate is 1.9%, indicating that it was not the method used but rather the nature of these isolates and their possession of characteristics of both *Shigella spp.* and *E. coli* that caused the uncertain assignments. The Random Forest method performed almost equally as well as the traditional laboratory tests and could be used for identification of the genus if whole genome data is available, although more isolates should be tested to validate this. Additionally, it would be useful to test the applicability of Random Forest for identification to species and serotype level. Furthermore, in a future study, the results of the traditional laboratory tests specifically can be associated with genetic variants. Consequently, if associated variants could be found, traditional tests could be omitted. This will save costs in workflows that already consist of draft genome sequencing of isolates for other purposes, for instance surveillance.

In addition to the methods using gene presence/absence and k-mers that were used in our study, other types of genetic variants can be used as input for microbial GWAS [31]. The k-mer approach used in this study is able to detect different genetic variants such as SNPs, indels, variable promotor regions and gene content simultaneously [32]. This indicates that adding purely SNP-based methods to the methods used is redundant as SNPs are already encompassed in the k-mer method performed. Another genetic variant that can be used in GWAS is based on De Bruijn Graphs. However, it is mainly based on the creation of overlaps of k-mers, therefore, it probably would not generate associations with symptoms or disease severity using the data from our study [33].

One of the strengths of our study was the availability of isolates representative of the population structure encountered in other western European countries, as well as the clinical data of the patients that they were infecting. Second, results of the traditional laboratory tests performed to determine the species of the bacteria were available for all isolates. Finally, another strength of our study is that several potential genetic variants were associated with the trait “genus”, and a sensitivity analysis was performed, both proving the suitability of the algorithms used.

Some considerations with regard to our study should be taken into account. The impact of several factors regarding host-variability is unknown, as the symptoms and severity of disease were characteristics of the patients and not directly of the bacterial isolates. First, the immune status of the patients was not taken into account because data was not available, although the need for correction of the effects of underlying disease was investigated. Second, the clinical characteristics used in our study were self-reported and not objectively measured, therefore subject to the judgment and memory of the patients. To overcome these difficulties of host-variability, an infection model can be used for future investigations into genetic factors of *Shigella* isolates that influence the disease severity of patients. Because *Shigella spp.* are host-adapted to humans only, recently developed human intestinal enteroids are more appropriate for this purpose than animal models [34]. Additionally, inconsistencies between the two scoring methods used were present (Fig. 1 and Additional file 4). Because each scale uses different criteria with different weighing for calculation of the score, patients can be classified in different severity classes depending on the severity scale used. Therefore, conclusions based on research into severity of gastro-intestinal infections in general are highly dependent on the chosen severity scale. To rule out this dependency, in this study, both scores were associated with genetic content separately. Another consideration was that genus level was associated as a characteristic, while other GWAS studies have concentrated on bacterial isolates of the same species [35, 36]. However, according to multiple research groups [3, 37, 38] *Shigella spp.* and *E. coli* should be considered as one species based on their genetic relatedness, if present, their differences are more phenotypical. Next to this, the number of isolates for *S. boydii* and *S. dysenteriae* in our study were inadequate with two and no isolates, respectively. However, we believe the total number of isolates to be adequate, as studies with similar sample sizes have been performed in the past in which genetic variation in pathogens was identified that had predictive value for the course of disease [29, 39]. Finally, the dataset used only contained isolates encountered in the Netherlands, resulting in a geographical biased set [40, 41]. Therefore, to avoid missing serotypes in future studies, the current dataset should be supplemented with isolates from other geographic areas.

## Conclusions

Using several microbial GWAS methods, genetic variants in *Shigella spp.* and EIEC that can predict specific symptoms or a higher disease severity were not found. In contrast to adjustment of the guidelines of STEC, genes or gene fragments that indicate higher risks for a more severe course of disease does not appear to exist for shigellosis, whether caused by *Shigella* or EIEC, using the dataset in our study. Therefore, the bacterial specific genes or gene fragments from patient isolates are not suitable to predict outcomes in individual patients or to use in development, prioritization and optimization of guidelines for control measures of shigellosis or EIEC. As GWAS in our study associated genetic fragments with genus, future studies can be performed in which GWAS could support the distinction of *Shigella spp.* from EIEC. Additionally, the prediction of results of traditional laboratory tests using draft genome sequences could be performed using GWAS. The results of these suggested follow-up studies could improve diagnostics and guidelines for control measures of shigellosis.

## Methods

### Bacterial isolates and clinical data

The data used in our study was collected during the Invasive Bacteria *E. coli-Shigella* Study (IBESS). IBESS was a cross-sectional study in the Netherlands, of which one of the aims was to fill the gap of knowledge about the incidence, clinical implications and impact on public health of infections caused by EIEC. During this study, in 2016 and 2017, EIEC and *Shigella* isolates were collected, together with epidemiological patient data (van den Beld et al., manuscript submitted). Isolates were identified using an identification scheme, using traditional laboratory tests as previously described [42]. In short, it consists of a Polymerase Chain Reaction (PCR) of the *ipaH* gene, followed by thorough phenotyping and classical *E. coli* and *Shigella* O-antigen serotyping by agglutination. The draft genome sequences of a set of 277 bacterial isolates, of which patient data was available, were used as genetic input data. The set comprises *S. sonnei* (*n* = 163), *S. boydii* (n = 1), *S. flexneri* (*n* = 77), EIEC (*n* = 30), provisional *Shigella* (*n* = 5), which are *Shigella* isolates with an undescribed serotype, and one isolate of which the distinction between *S. flexneri* and EIEC was unclear, using the traditional laboratory tests.

The clinical characteristics that were used in this GWAS study were symptoms and disease severity of patients infected with *Shigella spp.* or EIEC isolates included in IBESS. For all patients, a list of symptoms including abdominal pain, abdominal cramps, blood in stool, diarrhea, fever, headache, mucus in stool, nausea, and vomiting was available and was used as binary input (Additional file 4). Additionally, disease severity was calculated using two severity scales, both are modifications of the Vesikari scale, a widely used method in clinical studies [43]. These modifications, the MVS [44] and the modified score of de Wit et al. [45]*,* were both developed and validated for outpatient settings in high-resource areas. With these severity scores, lower scores indicate a milder course of disease [44, 45]. The calculated scores were stratified into scales representing mild, moderate and severe disease according to their own categorization and used as dichotomous input in the GWAS methods that were assessing the presence/absence of genes in this study (Additional file 4). For the de Wit severity score, a score of 0–3 is considered mild, 4–6 moderate and ≥ 7 severe [45]. The designers of the MVS score consider a score of 0–8 as mild, 9–10 as moderate and ≥ 11 as severe disease [44]. Alternatively, for the GWAS method that assessed k-mers, both severity scores were used as a continuous input.

Laboratories that participated in IBESS, reported other bacteria, viruses and parasites detected in the fecal samples by molecular, culture and microscopy methods as well (Additional file 4). Other pathogens were detected in 15.5% of the patients from whom the *Shigella spp.* and EIEC isolates were isolated that were used in this GWAS study. The statistical differences in symptoms and severity scores of patients with and without coinfections were assessed, in order to establish if these pathogens have impact on the symptoms experienced. If necessary, data about effects of underlying diseases of the patients were used as a correction. Additionally, the genus of the bacteria was used as directly derived characteristic to use as a control to verify the suitability of the methods used. The patient data used in the GWAS studies are depicted in Additional file 4.

### Genome sequencing and data preparation

DNA isolation and short-read Illumina sequencing was performed as described earlier [42]. For preparation of the genomes, an in-house assembly pipeline available at GitHub (https://github.com/Papos92/assembly_pipeline) was used. It consists of raw data quality assessment using FastQC v. 0.11.8 [46] and MultiQC v. 1.7 [47], read trimming using ERNE v. 2.1.1 [48], contamination filtering using CLARK v. 1.2.5.1 [49], contigs and scaffold assembly using SPAdes v. 3.10.0 [50], and assembly quality assessment using QUASTv. 4.4 [51]. Contigs smaller than 200 bp or with a coverage < 10 were filtered out. CheckM v. 1.0.11 [52] (taxonomy_wf: genus ‘Shigella’) was used for quality assessment, genome completeness and contamination checks of the assemblies. Isolates with completeness above 99% and a contamination below 2% were included for further analyses. Sequences of isolates are available from the Sequence Read Archive (SRA) with study number PRJEB32617 (https://www.ncbi.nlm.nih.gov/sra/), accession numbers are indicated in detail in Additional file 3.

Prokka v. 1.1 [53] was used without cleanup for annotation of the genomes. Gene presence/absence for all genomes was determined using Roary v. 3.12.0 [54], using a BLAST identity cutoff of 80% and with paralog splitting disabled. Phylogenetic trees based on core genome SNPs were constructed with Parsnp v.1.2 [55]. To include contextualization of the position of the isolates sequenced in this study relative to the main lineages of EIEC and *S. sonnei* and the phylogenetic groups (PG) of *S. flexneri* randomly selected genomes from each lineage or phylogenetic group were included in the phylogenetic tree [3, 19, 22, 56, 57]. Details of these representatives and their accession numbers are depicted in Additional file 5. Data was visualized using iTol v. 4.3 [58].

### GWAS using gene presence/absence of single genes

Scoary v. 1.6.16 [26] was used to associate gene presence and absence with the symptoms and severity of patients and the genus of the isolates, using a *p*-value cut-off of 0.5. Output was generated as a list of associated genes per characteristic with their best pairwise comparison *p*-values, sensitivity, and specificity. For each characteristic, as benchmark, a 1000 random datasets were created by shuffling the original traits randomly for a thousand times using a custom script [59]. For each symptom and severity scale, 1000 genes with the lowest ‘best pairwise p-value’ were used, this p-value takes population structure into account. The observed p-values of the traits were log transformed and plotted against the log transformed expected p-values of the permutation benchmark using a custom script [59]. For the characteristic ‘genus’, Benjamini-Hochberg’s method for multiple comparisons correction is used instead of pairwise p-values as the latter cannot be used to find genetic differences between the species and genera. Additionally, a sensitivity analysis including corrections for multiple testing and the population structure was performed. To assess the minimal number of isolates with gene presence that is needed to detect a significant association, the corrected p-values from the output for the association of genes with the characteristic “genus” were log transformed and plotted against the percentage of isolates in which the corresponding genes were present (Additional file 2).

### GWAS using gene presence/absence of multiple genes

Random Forest classification was executed using R v. 3.4.4 [60] and the randomForest package v. 4.6–14 [61]. The gene presence/absence table derived from Roary and the symptoms and severity of patients and the genus of the isolates were used as input. The dataset was divided over a test set and a training set. Potential class size differences were corrected by using two-thirds of the smallest class as the sample size to create models based on gene presence/absence of multiple genes in the training set, using 5000, 8000 and 10,000 trees respectively. The performance of these models was validated by predicting the outcome of each trait using the genomes of the isolates in the test set.

### GWAS using k-mers

To generate the k-mers that were associated with the characteristics, first, a population structure estimation was made using mash v. 2.0 [62]. Second, k-mer counting was performed using fsm-lite v. 2.0.3, and the optimal number of dimensions to use as co-factors in the analysis was determined [32, 63]. Subsequently, to estimate the effect of the k-mers on the severity scores and patient symptoms, Pyseer v. 1.1.2 was used with the following settings: a maximum of six dimensions, a filter *p*-value of 1E-8, a minimum allele frequency of 0.02 and a maximum allele frequency of 0.98.

The resulting k-mers were aligned using ClustalW v. 2.1, which resulted in one consensus sequence [64]. To identify the position of the k-mers in the genome, the resulting consensus sequence was aligned using the nucleotide Basic Local Alignment Search Tool (BLASTn) v. 2.8.1 with default settings [65]. To investigate whether the k-mer contained a promotor, BPROM was used [66].

In addition, to validate the association of the resulted consensus k-mer with the characteristics it was aligned against a BLAST database of all assembled genomes from this study, created using BLASTn v. 2.2.31+ [67]. Best scoring hits including bit-score were collected for all isolates, plotted against the severity score and a Kruskal-Wallis test was performed using GraphPad prism v. 7.04 (GraphPad Software, La Jolla California USA).

## Supplementary information

**Additional file 1. **Phylogenetic tree based on core genome SNPs. In this figure, the isolates from this study were placed in context by adding representative genomes from main lineages and phylogroups of EIEC, *S. flexneri* and *S. sonnei*.

**Additional file 2.** Sensitivity analysis of the characteristic “genus”. In this figure, the sensitivity analysis is visualized.

**Additional file 3.** Location of the consensus of k-mers associated with severity score of de Wit.

**Additional file 4.** Genomes and phenotypic data used in GWAS. The phenotypic patient data used for the GWAS, including accession numbers of the genomes of the infected isolates are depicted in this table.

**Additional file 5.** Representative genomes. The accession numbers and publications of the representative genomes used in this study are depicted in this table.

## Data Availability

Sequences of isolates were submitted to the European Nucleotide Archive (ENA, EMBL-EBI, Cambridge, United Kingdom) with study number PRJEB32617 (https://www.ebi.ac.uk/ena). The in-house assembly pipeline is available on Github: https://github.com/Papos92/assembly_pipeline. All scripts and commands used for data preparation and GWAS are available on zonodo (10.5281/zenodo.3626738). All other data generated or analyzed during this study are included in this published article and its supplementary files.

## Abbreviations

BLAST: Basic local alignment search tool
EIEC: Entero-invasive *Escherichia coli*
GWAS: Genome wide association studies
IBESS: Invasive Bacteria *E. coli-Shigella* Study
LRT: Likehood-ratio test
MVS: Modified Vesikari Score
NCBI: National Center of Biotechnology Information
OOB: Out-of-bag
PCR: Polymerase Chain Reaction
PG: Phylogenetic Group
RF: Random Forest
SNP: Single nucleotide polymorphism
ST: Sequence type
STEC: Shiga toxin- producing *Escherichia coli*
