# Supplementary figures and images for: Genome-wide association studies of Shigella spp. and Enteroinvasive Escherichia coli isolates demonstrate an absence of genetic markers for prediction of disease severity
Source: BMC Genomics. 2020 Feb 10;21:138. doi: 10.1186/s12864-020-6555-7 (PMC7011524; doi:10.1186/s12864-020-6555-7)

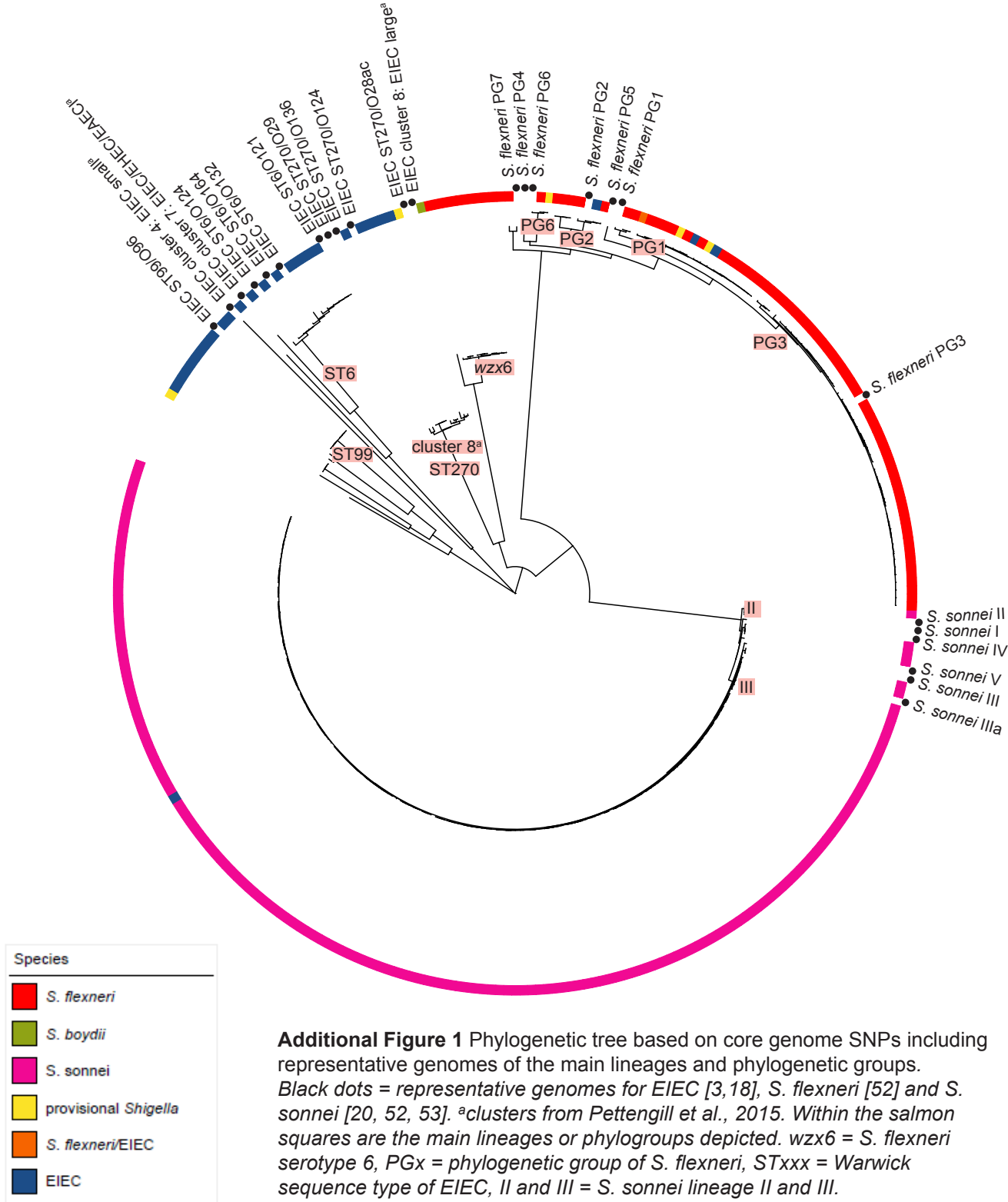

Supplement: Supplementary file 1 — Additional file 1. Phylogenetic tree based on core genome SNPs. In this figure, the isolates from this study were placed in context by adding representative genomes from main lineages and phylogroups of EIEC, S. flexneri and S. sonnei. [file 12864_2020_6555_MOESM1_ESM.pdf]
