# Supplementary material for: Genome-wide association studies of Shigella spp. and Enteroinvasive Escherichia coli isolates demonstrate an absence of genetic markers for prediction of disease severity
Source: BMC Genomics. 2020 Feb 10;21:138. doi: 10.1186/s12864-020-6555-7 (PMC7011524; doi:10.1186/s12864-020-6555-7)
Supplement: Supplementary file 2 — Additional file 2. Sensitivity analysis of the characteristic “genus”. In this figure, the sensitivity analysis is visualized. [file 12864_2020_6555_MOESM2_ESM.pdf]

Additional File 2

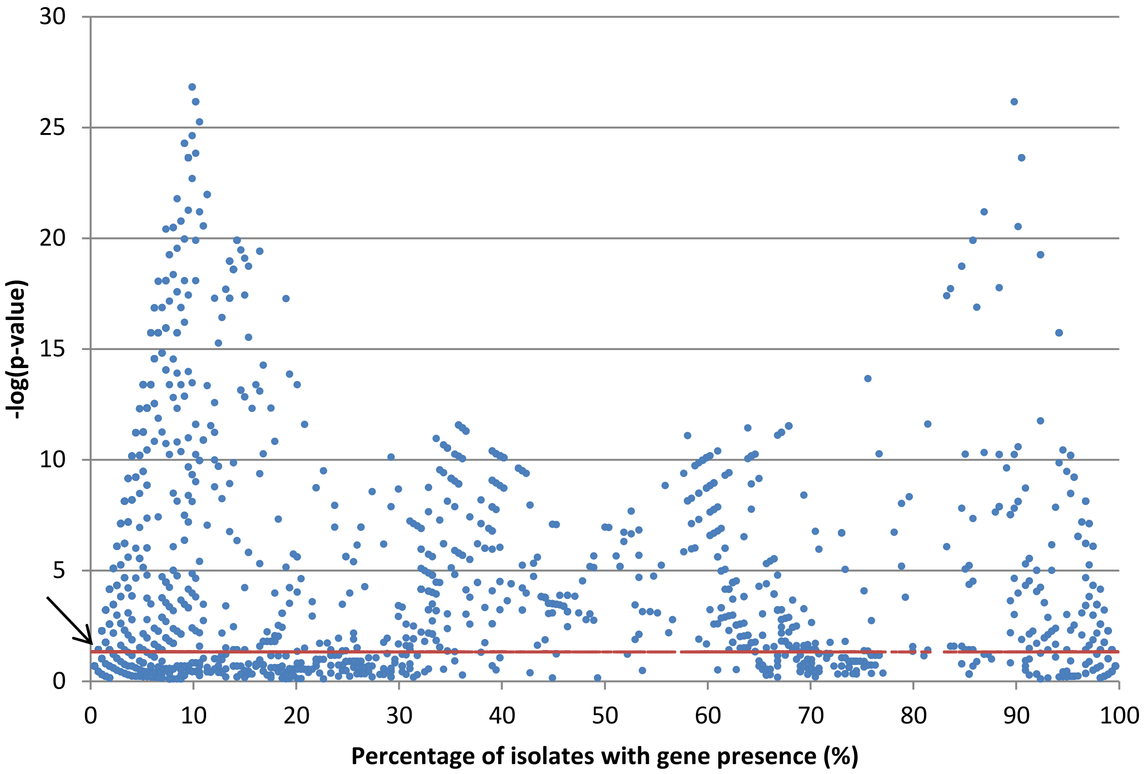

Additional File 2. Sensitivity analysis of the characteristic “genus”
Blue dots = log transformed p-values (corrected for multiple testing and population structure) plotted against the percentage of isolates with gene presence from total number of isolates (n = 277). Red horizontal line = significance level, -log p = 1.3 (p=0.05). Black arrow = value above significance with lowest percentage of isolates with gene presence (0.7%).
