# Supplementary material for: Genome-wide association studies of Shigella spp. and Enteroinvasive Escherichia coli isolates demonstrate an absence of genetic markers for prediction of disease severity
Source: BMC Genomics. 2020 Feb 10;21:138. doi: 10.1186/s12864-020-6555-7 (PMC7011524; doi:10.1186/s12864-020-6555-7)
Supplement: Supplementary file 3 — Additional file 3. Location of the consensus of k-mers associated with severity score of de Wit. [file 12864_2020_6555_MOESM3_ESM.pdf]

Additional File 3

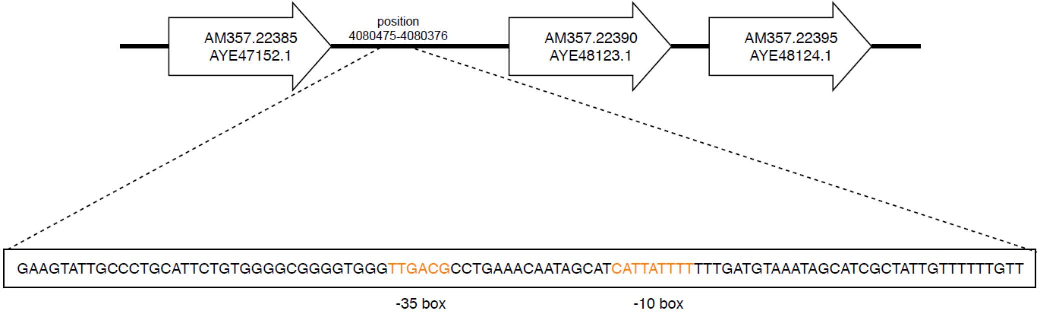

Additional File 3. Location of the consensus of the k-mers associated with the severity score of de Wit

Genome of Shigella sonnei, CDC strain AR300 (accession number: CP032523.1), including location -10 and -35 box of a potential promoter in red.
